# Adhesion Development and the Expression of Endothelial Nitric Oxide Synthase

**DOI:** 10.1155/S1064744901000199

**Published:** 2001

**Authors:** David M. Svinarich, Fadi M. Zaher, Lena Holmdahl, Nasser Chegini, Bernard Gonik, Michael P. Diamond

**Affiliations:** 1 Department of Obstetrics and Gynecology Wayne State University School of Medicine C.S. Mott Center 275 E. Hancock Ave. Detroit MI 48201 USA; 2 Department of Obstetrics and Gynecology University of Florida Gainesville FL USA; 3 Department of Surgery Sahlgrenske University Hospital University of Göteborg Göteborg Sweden

## Abstract

*Objective:* This study was conducted to determine whether nitric oxide (NO), a potent vasodilator and inhibitor of thrombus formation, is involved in the formation and maintenance of adhesions.

*Methods:* Skin, subcutaneous tissues, peritoneum and adhesions were collected from surgical patients and total RNA was isolated. Quantitative reverse transcription polymerase chain reaction (QRT-PCR) was performed to quantitate endothelial nitric oxide synthase (eNOS) and β-actin mRNA levels.

*Results:* eNOS mRNA levels for skin, subcutaneous tissue, peritoneum and adhesions were ≤ 3.12 × 10^-4^, ≤ 3.12 × 10^-4^, 6.24 × 10^-4^ and 2.5 × 10^-3^ attomoles/μl, respectively. β-actin mRNA levels for all tissues were between 1.25 × 10^-1^ and 6.25 × 10^-2^ attomoles/μl.

*Conclusion:* eNOS mRNA can be identified in tissue adhesions, and may therefore play a role in adhesion formation and maintenance.


*Infect Dis Obstet Gynecol 2001;9:113–116*

### Adhesion development and the expression of endothelial nitric oxide synthase

**David M. Svinarich[1], Fadi M. Zaher[1], Lena Holmdahl[3], Nasser Chegini[2], Bernard Gonik[1] and Michael P. Diamond[1], for the PHAMUS group**

*[1]Department of Obstetrics and Gynecology, Wayne State University School of Medicine, Detroit, MI*
*[2]Department of Obstetrics and Gynecology, University of Florida, Gainesville, FL*
*[3]Department of Surgery, Sahlgrenske University Hospital, University of Göteborg, Göteborg, Sweden*
*The Peritoneal Healing and Adhesion Multi-University Study (PHAMUS) Group: Chegini N, Bennet B, Goldberg E, Kosteos K, McLean F, Peck L, Zhao Y, Leach R, Saed G, Munkarah A, Sorokin Y, Yelian F, Zhang W, Collins K, Holmdahl L, Falk P, Hedgren M, Ivarsson ML, Palmgren I*

***Objective:*** This study was conducted to determine whether nitric oxide (NO), a potent vasodilator and inhibitor of thrombus formation, is involved in the formation and maintenance of adhesions.
***Methods:*** Skin, subcutaneous tissues, peritoneum and adhesions were collected from surgical patients and total RNA was isolated. Quantitative reverse transcription polymerase chain reaction (QRT-PCR) was performed to quantitate endothelial nitric oxide synthase (eNOS) and β-actin mRNA levels.
***Results:*** eNOS mRNA levels for skin, subcutaneous tissue, peritoneum and adhesions were $\leq 3.12 \times 10^{-4}$, $\leq 3.12 \times 10^{-4}$, $6.24 \times 10^{-4}$ and $2.5 \times 10^{-3}$ attomoles/μl, respectively. β-actin mRNA levels for all tissues were between $1.25 \times 10^{-1}$ and $6.25 \times 10^{-2}$ attomoles/μl.
***Conclusion:*** eNOS mRNA can be identified in tissue adhesions, and may therefore play a role in adhesion formation and maintenance.

Key words: ADHESIONS, eNOS, TRANSCRIPTIONAL REGULATION, QRT-PCR

Adhesions can result from surgically induced trauma to the peritoneum or through inflammatory and infection processes arising from such conditions as endometriosis, pelvic inflammatory disease (PID) and appendicitis. Intraperitoneal adhesions within the pelvis often result in infertility, recurrent pelvic pain, small bowel obstruction and fixation as well as difficult re-operative surgery. These factors contribute substantially to patient morbidity and result in a dramatic escalation in health care costs[1,2]. Consequently, there is a pressing medical need to more fully define the pathophysiology underlying adhesion development.

The stimuli responsible for initiating adhesion formation are incompletely understood. However, ischemia and hypoxia are recognized as important initiating events. Likewise, the infection-mediated cascade of inflammatory byproducts may play a role in adhesion formation. Vascular infiltration within fibrinous adhesive bands has also been recognized as an important step in the formation of some adhesions[3]. The mediators necessary for the development and

Supported by Genzyme Corporation, Cambridge, Massachusetts.
Presented at the 46th Annual Meeting of the Society for Gynecologic Investigation, Atlanta, GA, March 10-13, 1999.
Correspondence to: David M. Svinarich, PhD, Department of Obstetrics and Gynecology, Wayne State University School of Medicine, C.S. Mott Center, 275 E. Hancock Ave., Detroit, MI 48201. E-mail: d.svinarich@wayne.edu maintenance of perfusion within adhesions are at present unknown. However, a probable candidate responsible for maintaining vascular homeostasis, preventing thrombus formation and inhibiting vasoconstriction is nitric oxide (NO).

NO is a potent and labile vasodilator that is produced by three genetically distinct isoforms of nitric oxide synthase including neuronal (nNOS), inducible (iNOS) and constitutive NOS (cNOS)[4–6]. Endothelial nitric oxide synthase (eNOS), a constitutive form of NOS, plays a vital role in the regulation and maintenance of vascular smooth muscle tone. eNOS also inhibits platelet aggregation and attenuates the action of certain vasoconstrictors[7]. eNOS transcription is known to be regulated by hormones, shear stress, pro-inflammatory cytokines and hypoxia[8–11]. Wound healing, a process analogous in many respects to adhesion formation, has been associated with increased NO levels[12,13].

This pilot study was conducted to determine whether transcriptional expression of eNOS could be identified in intraperitoneal adhesion tissues. Specifically, the presence and level of eNOS mRNA transcripts were determined within adhesions and analyzed for comparison in skin, subcutaneous tissue and normal peritoneum by quantitative reverse transcription polymerase chain reaction (QRT-PCR).

### METHODS

### Sample collection and RNA isolation

Skin, subcutaneous tissue, peritoneum and intra-abdominal adhesions ($n$ = 3 per tissue type) were obtained from surgical patients undergoing gynecologic procedures and stored at −70°C. No attempts were made to clinically establish the cause or longevity of the adhesions. Tissue samples were pulverized under liquid nitrogen. Total RNA was isolated using the Trizol® reagent (GIBCO BRL, Gaithersburg, MD), in accordance with the manufacturer's instructions. RNA was rendered free of contaminating genomic DNA by treatment with RQ1 DNase (5 U/100 mg of tissue), for 90 min at room temperature (Promega, Madison, WI). RNA integrity was confirmed by gel electrophoresis and quantitated spectrophotometrically at 260 nm and 280 nm. RNA was subsequently stored at −70°C in the presence of the ribonuclease inhibitor RNasin (40 U/sample) (Promega, Madison, WI). All specimens were collected with prior approval and in accordance with guidelines established by the Wayne State University, University of Florida and the University of Goteborg Institutional Review Boards.

### Quantitative reverse transcription polymerase chain reaction

QRT-PCR is a highly sensitive technique that enables the amplification and quantitation of specific cDNA transcripts. A non-homologous DNA fragment of known concentration and bearing the same eNOS or β-actin primer sites served as the competitive internal standard or MIMIC. Equimolar concentrations of the cDNA of interest and MIMIC will yield PCR products with equivalent band intensities, thereby enabling quantitation and comparison among groups. β-actin mRNA concentrations, as determined by QRT-PCR, were used to control for minor differences in the concentration of total RNA used in all reactions.

Total RNA (1 μg) was reverse transcribed at 70°C for 1 h in a 50 μl reaction volume containing rTth DNA polymerase (5 U), dNTPs (150 μM), $Mn(OAc)_2$ (2.0 mM), β-actin or eNOS anti-sense and sense primers (2.5 mM), β-actin MIMICs ($5 \times 10^{-1}$ to $6.25 \times 10^{-2}$ attomoles) or eNOS MIMICs ($5 \times 10^{-3}$ to $3.12 \times 10^{-4}$ attomoles), and 1X EZ Buffer (Perkin Elmer Corp., Applied Biosystems Division, Foster City, CA). The resultant cDNA was denatured at 95°C for 2 min and used directly for QRT-PCR. DNA was denatured at 95°C for 45 s, annealed at 72°C for 45 s and amplified at 60°C for 45 s, for 35 cycles. Amplification was concluded with a single terminal extension period at 72°C for 15 min. Cycle number and cDNA concentration were adjusted so that amplified products remained within the linear range of the PCR reaction. PCR amplification was conducted on a Perkin Elmer DNA Thermal Cycler 480 (Perkin Elmer Corp., Norwalk, CT). Human-specific β-actin and eNOS amplimers and MIMICs were synthesized commercially using sequence data available in the public domain (Integrated DNA technologies, Inc., Coralville, IA). Amplimers used in this study were designed to span intronic regions to detect the presence of contaminating genomic DNA. PCR products were resolved electrophoretically and size was confirmed by comparison with a molecular size standard (λ DNA, Pvu II digest). Band intensities were determined by scanning laser densitometry.

### RESULTS

QRT-PCR amplification of skin, subcutaneous tissue, peritoneum and adhesions yielded anticipated products of 309 bp and 537 bp for the β-actin cDNA and MIMIC and 551 bp and 441 bp for the eNOS cDNA and MIMIC, respectively. β-actin mRNA concentrations for all tissues were between $1.25 \times 10^{-1}$ and $6.25 \times 10^{-2}$ attomoles/μl (Figure 1). Representative eNOS mRNA concentrations for skin, subcutaneous tissue, peritoneum and adhesions were $\leq 3.12 \times 10^{-4}$, $\leq 3.12 \times 10^{-4}$, $6.24 \times 10^{-4}$ and $2.5 \times 10^{-3}$ attomoles/μl, respectively (Figure 2). Similar levels of mRNA transcription were seen in all tissue samples studied. The level of eNOS mRNA found in adhesions was approximately 4-fold greater than that found in intact peritoneal tissue and approximately 8-fold greater than eNOS mRNA levels found in either skin or subcutaneous tissue.

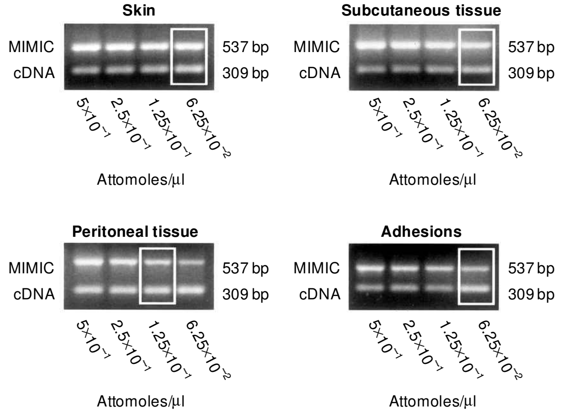

**Figure 1** Quantitation of β-actin mRNA in skin, subcutaneous tissue, peritoneum and adhesions by quantitative reverse transcription polymerase chain reaction (QRT-PCR). β-actin mRNA from skin, subcutaneous tissue, peritoneum and adhesions was quantitated by co-amplification with internal standards (MIMICs) of known concentration ($6.25 \times 10^{-2}$ to $5 \times 10^{-1}$ attomoles/μl). Amplification yielded anticipated β-actin cDNA products of 309 base pairs (bp) and β-actin MIMIC products of 537 bp. PCR product bands of equivalent intensity are indicated by a box, for each tissue type. The corresponding concentration of mRNA, in attomoles/μl, is shown below

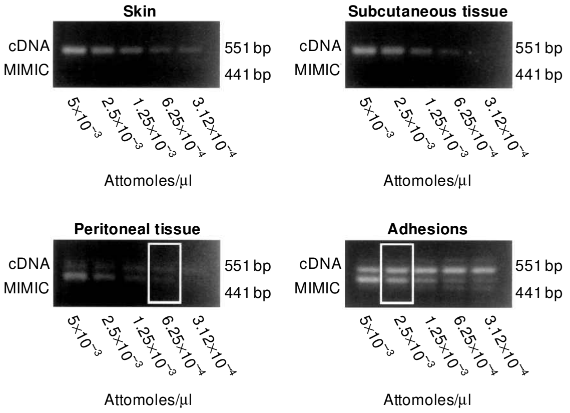

**Figure 2** Quantitation of endothelial nitric oxide synthase (eNOS) mRNA in representative skin, subcutaneous tissue, peritoneum and adhesions by quantitative reverse transcription polymerase chain reaction (QRT-PCR). eNOS mRNA from skin, subcutaneous tissue, peritoneum and adhesions was quantitated by co-amplification with internal standards (MIMICs) of known concentration ($3.12 \times 10^{-4}$ to $5 \times 10^{-3}$ attomoles/μl). Amplification yielded predicted eNOS cDNA products of 551 base pairs (bp) and eNOS MIMIC products of 441 bp. PCR product bands of equivalent intensity are indicated by a box for each tissue type except in instances where mRNA concentrations are below the lowest MIMIC concentration used. The corresponding concentration of mRNA, in attomoles/μl, is shown below

### DISCUSSION

The development of intraperitoneal adhesions following infection, pelvic surgery or *de novo* are prevalent gynecologic occurrences. Collectively, intraperitoneal adhesions are a significant cause of recurrent pelvic pain, small bowel fixation and obstruction, difficult re-operative surgery and infertility. Numerous adjuvants including fibrinolytic agents, anticoagulants, anti-inflammatory agents, antibiotics and both chemical and physical barriers have, with a limited effect, been used in an effort to preclude the development of adhesions[3]. For the most part these approaches have been empirically derived. Better understanding of the pathophysiology underlying adhesion formation, and how this may differ based on the inciting etiology, is necessary for the development of efficacious interventions. Vascular support of formed adhesions via eNOS or other regulators is an area of research that deserves attention.

The data presented in this pilot study demonstrate that adhesions transcriptionally express eNOS. Furthermore, eNOS mRNA levels are substantially greater in adhesions than in either skin, subcutaneous tissue or normal peritoneum. This suggests that NO may be an important modulator of adhesion formation and maintenance. These observations are supported by studies on general wound healing, a process that is analogous in many respects to the formation of adhesions. NO expression is increased during wound healing and is necessary for both wound closure and the maintenance of sufficient tensile strength[11–14]. Work is currently underway to examine eNOS expression in adhesions formed via different pathophysiologic events (e.g. after PID, surgery, endometriosis), and from various anatomic sites within the pelvis. Timed studies examining the evolution of adhesions (and their modulators) are also needed. These types of investigation, focused on eNOS expression, may reveal new targeted therapeutic modalities aimed at the regulation of perfusion or vasculogenesis at sites of tissue injury.

### REFERENCES

1. Diamond MP, Daniell JF, Martin DC, *et al.* Tubal patency and pelvic adhesions at early second-look laparoscopy following intraabdominal use of the carbon dioxide laser: initial report of the intra-abdominal laser study group. *Fertil Steril* 1984; 42:717–23
2. Diamond MP. Surgical aspects of infertility. In Sciarra J, ed. *Gynecological Obstetrics*. Philadelphia: Harper & Row, 1988;5:1–23
3. Gutmann J, Penzias AS, Diamond MP. Adhesions in reproductive surgery. In Wallach EE, Zacur HA, eds. *Reproductive Medicine and Surgery*. St Louis, Missouri: Mosby-Year Book, Inc, 1995:681–93
4. Bredt DS, Hwang PM, Glatt CE, *et al.* Cloned and expressed nitric oxide synthase structurally resembles cytochrome P-450. *Nature* 1991;351:714–18
5. Lowenstein CJ, Glatt CS, Bredt DS, Snyder SH. Cloned and expressed macrophage nitric oxide synthase contrasts with brain enzyme. *Proc Natl Acad Sci USA* 1992;89:6711–15
6. Lamas S, Marsden PA, Li GK, *et al.* Endothelial nitric oxide synthase: molecular cloning and characterization of distinct constitutive enzyme isoform. *Proc Natl Acad Sci USA* 1992;89:6348–52
7. Marletta MA. Nitric oxide synthase structure and mechanism. *J Biol Chem* 1993;268:12231–4
8. Liao QP, Buhimschi IA, Saade G, *et al.* Regulation of vascular adaptation during pregnancy and postpartum: effects of nitric oxide inhibition and steroid hormones. *Hum Reprod* 1996;11:2777–84
9. Uematsu M, Ohara Y, Navas JP, Nishida K. Regulation of endothelial cell nitric oxide synthase mRNA expression by sheer stress. *Am Phys Soc* 95;269:1371–8
10. Lamas S, Michel T, Brenner BM, Marsden PA. Nitric oxide synthesis in endothelial cells: evidence for a pathway inducible by TNF-α. *Am Phys Soc* 1991;261:634–41
11. Seligman SP, Nishiwaki T, Kadner SS, *et al.* Hypoxia stimulates ecNOS mRNA expression in differentiated human trophoblasts. *Ann NY Acad Sci* 1997;828:180–7
12. Schaffer MR, Tantry U, Gross SS, *et al.* Nitric oxide regulated wound healing. *J Surg Res* 1996; 63:237–40
13. Schaffer MR, Tantry U, van Wesep RA, Barbul A. Nitric oxide metabolism in wounds. *J Surg Res* 1997;71:25–31
14. Yamasaki K, Edington HD, McClosky C, *et al.* Reversal of impaired wound repair in iNOS-deficient mice by topical adenoviral-mediated iNOS gene transfer. *J Clin Invest* 1998;101:967–71

RECEIVED 11/28/00; ACCEPTED 03/07/01
